# G-Ratio Commentary—Why You’ve Been Doing It Wrong

**DOI:** 10.1080/17590914.2025.2486962

**Published:** 2025-04-07

**Authors:** R. Douglas Fields, Dipankar J. Dutta

**Affiliations:** a The Nervous System Development and Plasticity Section, Eunice Kennedy Shriver National Institute of Child Health and Human Development, Bethesda, MD, USA; b Section on Quantitative Imaging and Tissue Sciences, Eunice Kennedy Shriver National Institute of Child Health and Human Development, Bethesda, MD, USA; c Gladstone Institutes, University of California at San Francisco, San Francisco, CA, USA

The g-ratio is fundamental in all morphological studies of myelin to quantify the relative thickness of the myelin sheath on axons, but the relationship of g-ratio to axon diameter is unclear. The g-ratio is calculated simply by taking the ratio of the diameter of the myelinated fiber to the diameter of the axon inside the myelin sheath. This results in a mean value of about 0.65 for axons in the PNS (Rushton, 1951) and about 0.77 in the CNS (Waxman & Bennett, 1972), which theoretically provides optimal conduction velocity regardless of axon diameter (Chomiak & Hu, 2009). Axons tend to be smaller diameter with thinner myelin in the CNS than in the PNS and thus have slightly larger g-ratios than in the PNS. There are, however, several ambiguities concerning g-ratio relationships, which have persisted since the inception of this measurement. Alexander Gow confronts this quandary in a new article published in this issue (Gow, 2025) and concludes that the most widely used approach of plotting g-ratio over axon diameter is inappropriate.

When g-ratios are plotted against axon diameter a linear relation with a positive slope is typically obtained, such that larger caliber axons have comparatively larger g-ratios (proportionately thinner myelin sheaths). An example from the authors of this commentary from one of their previously published studies on adult mouse optic nerve is shown in Figure 1 (Dutta et al., 2018). If there were no slope of g-ratio to axon diameter, there would be no need for such plots: the myelin thickness could be reported simply as the mean g-ratio for all axons sampled.

**Figure 1.**
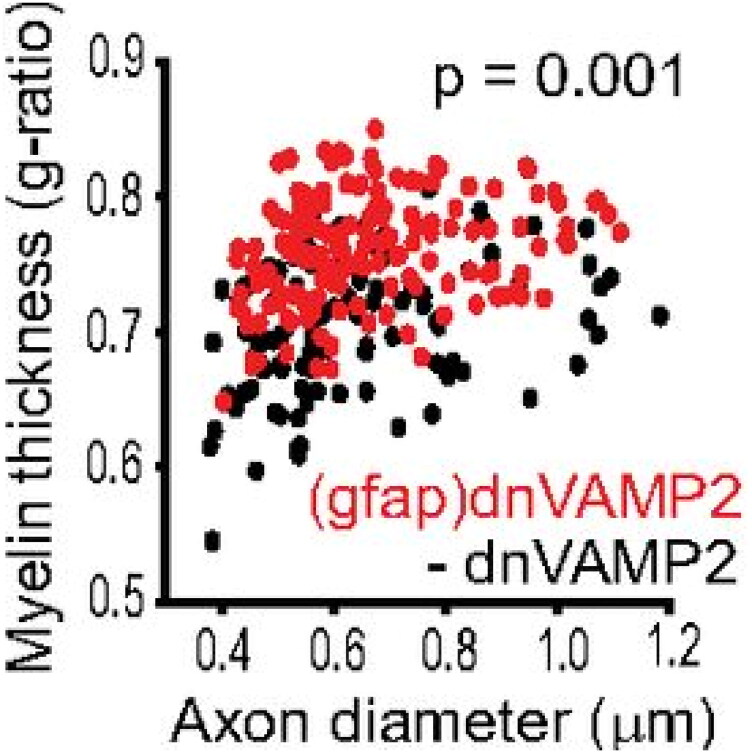
G-ratio of axons in adult mouse optic nerve expressing a transgene to reduce vesicle release from astrocytes (gfap dnVAMP2, in red), or not (-dnVAMP2, in black) showing the relation between g-ratio and axon diameter. Note the positive slope in both experimental and control groups. The myelin sheath thickness is reduced when exocytosis from astrocytes is decreased (higher g-ratio, in red). Reprinted from Dutta et al. (2018).

This relationship between g-ratio and axon caliber raises several questions. Why does this relation have a positive slope? What is the biological significance of differences in slope of this regression in different nerves or tracts or under different experimental conditions or pathological states?

According to this new study, the positive slope between g-ratio and axon diameter results from a combination of measurement and sampling artifacts. Several factors that contribute to a positive slope of g-ratio against axon core diameter are identified and explored by mathematical simulation in the study, most of which Gow finds are avoided by plotting g-ratio against total fiber diameter. (The total fiber diameter is the entire girth of the fiber including its myelin sheath.) This results in a slope of near zero; that is, the g-ratio is invariant over the full range of fiber diameters in a nerve or tract.

This finding, however, raises the issue of which relationship is biologically correct: the zero slope found when g-ratio is plotted against fiber diameter, or the positive slope obtained when g-ratio is plotted against axon diameter? Gow addresses this by using statistical analysis to evaluate how well sampled data from adult mouse optic nerve (Dupree et al., 2015) fit a regression, be it a linear regression or log transformed regression, against fiber diameter and axon diameter. The results show that plots of g-ratio against fiber diameter have significantly smaller residuals; i.e., less variation around the regression line. On this basis, the more accurate relationship is when g-ratio is plotted against total fiber diameter, and thus the myelin thickness relative to total fiber diameter is a constant.

To account for the common finding that there is a positive slope of g-ratio over axon diameter that becomes shallower for large caliber axons, simulations are used to assess the influence of different parameters on the curve fitting and correlations among the pertinent anatomical features. The analysis reveals that the linear relationship and y-intercept in plots of g-ratio over axon diameter are distorted by a number of sampling defects and measurement errors. Notably, the rank order of axon core diameters and fiber diameters (which includes the myelin sheath thickness) can differ, and this will contribute to distortion of the plots of g-ratios against axon caliber. G-ratios plotted against total fiber diameter fit a linear relationship better; i.e., with less variation around the regression line, than plots against axon diameter, and the y-intercept passes through the origin, which is a biological imperative (no myelin on axons of 0 diameter). This direct proportionality suggests a fundamental biological property relating myelin sheath thickness to fiber diameter, which is obscured when axon diameter is used as the independent variable in plots of g-ratio. Gow proposes that this is a fundamental biological relationship, which he terms the “axomyelin unit.”

## G-Ratio Distortions from Data Sampling Deficiencies

As a practical matter, how the g-ratios are determined is another factor contributing to the regression fits of g-ratio against axon caliber. Experimental errors are additive and random sampling effects differ with the size of the sampled population of axons and with the resolution of the measurements. Simulations show that each of these factors contributes to distorting the g-ratio when plotted against axon diameter. One of the sampling problems is that sample sizes differ for different sized subpopulations of axons in a large heterogeneous nerve or tract, typically with fewer large diameter axons. This can distort regression fits by unbalanced weighting across axon diameters and result in outlier points having an excessive influence on the linear curve fit. These effects are minimized by using total fiber diameter as the independent variable.

A second factor arises because measurements of g-ratio are often made near the limit of precision of the measurement method. The resolution limit of measurements can introduce different magnitudes of measurement error for axons of different calibers in the sampled population, and light-level microscopy and electron microscopy have vastly different limitations in this regard. Plotting g-ratios over total fiber diameter yields more accurate measurements that reduce the non-linearity and variation from the regression line, and reduce or eliminate the positive slope that occurs when plotting g-ratio against axon diameter.

Clearly, g-ratio measurement errors will differ considerably between light-level microscopy and electron microscopy, but each approach has different limitations that distort the data. Although the spatial resolution using electron microscopy is far superior to light microscopy, inadequate and inappropriate sampling become greater limitations with electron microscopy. This is because fewer fibers can be measured by electron microscopy from more spatially restricted anatomical regions. To an even greater extent than with light microscopy, which analyzes a larger field of view, fiber diameters in the population of axons are not sampled adequately or in equal numbers by electron microscopy. Also, the considerable shrinkage of tissue caused by fixation and embedding tissue will affect g-ratio measurements, and the fixation and embedding techniques vary widely in different light and electron microscopic studies. Tissue shrinkage of up to 65% has been reported in white matter during sample preparation (Skoven et al., 2023).

A major difficulty in electron microscopy is pseudo-replication. This results from experimental designs that measure many myelin profiles and then take each axon measurement as n (sample number) in statistical calculations. This artificially increases sample size and distorts statistical analysis in tests comparing different experimental groups, by violating the critical assumptions of independent sampling among groups in statistical tests such as t-tests and ANOVAs.

This problem applies to all quantitative electron microscopy studies, but this error is particularly persistent in published studies of myelin thickness and plasticity of node of Ranvier morphology. This pseudo-replication mistake greatly increases the error of concluding that there are differences among groups when there are in fact, no differences (false positives), simply because the ‘sample size’ used in the calculations is enormous (and erroneous). The results of published studies using this approach must be regarded as suspect.

This pseudo-replication error is minimized by more appropriate sampling designs that calculate averages of myelin or node of Ranvier measurements per microscope field, to adequately sample within-field variance, and use this value as a sample of n = 1, rather than taking every axon as an independent sample and using it as n (sample size) in the statistical calculation. Multiple fields of view are sampled from all experimental conditions to assess the within animal and between group variation. For example, in the study above by Dutta et al. (2018) nodal gap lengths and g-ratios were measured in thousands of optic nerve axons, but the mean value of gap length for all the nodes of Ranvier and g-ratios for all axons in each confocal or electron microscope field were averaged, and that value used as ‘n = 1’ for hypothesis testing statistics; e.g., n = 45 for wild-type mice (Dutta et al., 2018; Figure 3I).

The difficulties that arise from expressing g-ratios as a function of axon diameter is pertinent to new approaches of estimating axon diameters and g-ratios from magnetic resonance imaging (MRI) (Assaf & Basser, 2005; Assaf et al., 2008). These approaches are based on differences in proton diffusion in brain tissue, which differ within an axon and its myelin sheath. The myelin sheath has minimal diffusion of protons relative to the intracellular and extracellular compartments. Advances in MRI combine diffusion metrics with myelin-sensitive imaging to compute an ‘aggregate g‐ratio’ for entire fiber tracts. However, these ‘g-ratio weighted’ maps are subject to assumptions about myelin water fraction and axon density, which can distort absolute g-ratio estimates (Campbell et al., 2018). These imaging methods (Caminiti et al., 2009; Dyrby et al., 2018; Sepehrband et al., 2016; Tomasi et al., 2012) will necessarily generate g-ratio estimates that are based on the axon diameter, not the total fiber diameter, and will therefore result in g-ratio relationships with positive slopes against diffusion estimates of axon diameter.

## Limitations and Open Questions

A limitation of the study is that the analysis is based on data from one study on mouse optic nerve (Dupree et al., 2015), with the assumption that the measurement and sampling limitations in this dataset are representative of all other datasets performed on different tissues and in different experiments. As a practical matter, g-ratio measurements differ slightly in different studies; for example, the mean g-ratio for adult mouse optic nerve axons is smaller in the example provided here, about 0.72 (Dutta et al., 2018), than in the data used in Gow’s analysis of about 0.85. In part this reflects the range of axon diameters used for regression and mean g-ratio determination in these two studies. Axons with diameters up to about 3.5 µm are used in the dataset analyzed in Gow’s study, but axon size was limited to those smaller than 1.2 µm in the example from Dutta et al. (2018). The data were truncated in the Dutta et al., study because the small number of very large diameter axons in the optic nerve unbalances the sampling and skews the regression toward higher g-ratios. Irrespective of such variation in practice among different studies, the findings from Gow’s analysis pertain, and the conclusions are supported by the mathematical simulations.

Another consideration in the conclusion that there is a fixed myelin to fiber diameter relationship regardless of fiber diameter, is that the myelin sheath thickness can change. This occurs not only in pathological conditions and during development, but also in response to functional experience (Fields, 2015). Action potential firing activity can alter myelin sheath thickness on specific axons through the release of axo-glial signaling molecules and thereby modulate conduction velocities to achieve optimal spike time arrival at the target (Wake et al., 2011). Even in mature myelin the sheath can be dynamically thinned or thickened by a treadmilling mechanism regulating attachment of paranodal loops of myelin to the axon (Fields & Dutta, 2019).

Importantly, both myelin sheath thickness and axon diameter can differ or change independently from each other by different biological processes to alter the g-ratio. Changes in axon diameter that are independent of myelin sheath thickness are especially pertinent in pathological conditions that can cause shrinkage or swelling of axons and thus change the g-ratio and its relationship to axon caliber. Diseases or experimental treatments can also have selective effects on subpopulations of small or large diameter axons, or on the loss of axons, which will alter the g-ratio regression and mean g-ratio value. This is the primary motivation for inspecting axon diameter size-frequency distributions and g-ratios of axons of different calibers in experimental studies.

**Figure 2.**
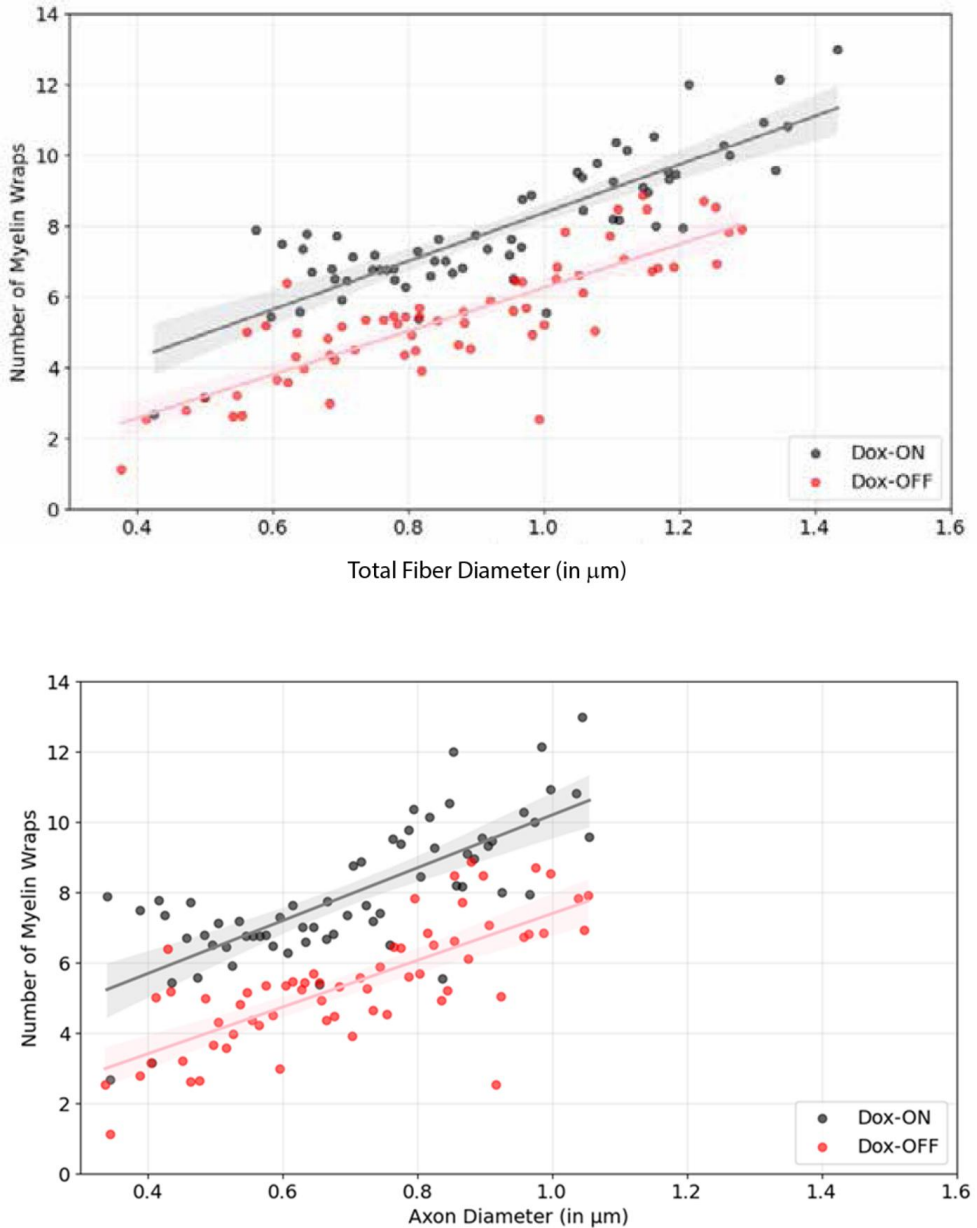
In contrast to g-ratios, there is a linear relation between the number of myelin wraps and axon caliber with a positive slope when plotted against either fiber diameter (above) or axon diameter (below) in both experimental (Dox off) and control (Dox on) conditions in adult mouse optic nerve. Data are from experiments by Dutta et al. (2018), which were not published previously, and can be compared with g-ratio plots from the same study in Figure 1.

Finally, although g-ratio is by far the most widely used approach to measuring myelination, other methods may yield different relationships of myelin thickness to axon fiber diameter. Myelin is not secreted; it is wrapped around axons by individual layers of compacted cell membrane, which increase the myelin sheath thickness incrementally by the same amount. If myelin in the study by Dutta et al. (2018) is assessed by counting the number of myelin wraps on optic nerve axons in adult mouse, a linear relation between myelin thickness yields a positive slope regardless of whether the data are plotted over axon diameter or total fiber diameter (Figure 2). The interpretation of this difference from using g-ratios is not clear; however, this is of limited practical value because g-ratio is by far the most common method of assessing myelin sheath thickness. A larger practical issue is that if plotting g-ratio against total fiber diameter becomes the norm, how can new results be compared with the past body of research?

## Summary

This new study shows that plotting g-ratios against total fiber diameter rather than axon diameter is the most appropriate method, and that when done in this way, the g-ratio is constant for axons of different fiber diameters. Adopting this practice will increase reliability of data on myelination in the wide range of applications where myelin sheath thickness differs or is altered by treatment.
